# Association Between Sleep Debt and Mental Well-Being in School-Aged Children and Adolescents in China: A Multi-Province, Cross-Sectional, Observational Study

**DOI:** 10.3390/healthcare14162503

**Published:** 2026-08-12

**Authors:** Nannan Wen, Xia Zhong, Peijin Hu, Jun Ma, Ning Li, Jiayi Guo, Dachao Zhang, Ziyue Sun, Yang Yang, Kaiheng Zhu, Yajie Wang, Jing Li, Yi Song

**Affiliations:** 1 College of Physical Education and Health, Heze University, Heze 274015, China; 2 Luoyang Orthopedic-Traumatological Hospital of Henan Province (Henan Provincial Orthopedic Hospital), Luoyang 471000, China; 3 School of Physical Education and Sport, Henan University, Kaifeng 475001, China; 4 Institute of Child and Adolescent Health, School of Public Health, Peking University, Beijing 100191, China; 5 National Health Commission Key Laboratory of Reproductive Health, Peking University, Beijing 100191, China; 6 Department of Physical Education, Peking University, Beijing 100871, China

**Keywords:** children and adolescents, mental well-being, sleep debt, weekend sleep patterns, nap duration, sleep deprivation

## Abstract

**Highlights:**

**What are the main findings?**

- Sleep debt was associated with greater odds of lower mental well-being among school-aged children and adolescents in China, with each quartile increase in sleep debt corresponding to 9.5% higher odds.
- The association was numerically larger among girls and rural students and was further modified by weekend sleep patterns and age-specific nap duration among adolescents aged 15–17 years.

**What are the implications of the main findings?**

- Sleep debt may represent a modifiable behavioral marker for identifying school-aged children and adolescents at increased risk of lower mental well-being.
- Pending subsequent verification of causal associations, promoting weekday sleep sufficiency, maintaining relatively regular weekend sleep schedules, and optimizing age-appropriate nap practices may support mental well-being promotion in school settings.

**Abstract:**

**Background/Objectives:** The association between sleep deprivation and mental well-being in school-aged children and adolescents has been well documented, but the specific relationship between sleep debt and mental well-being remains unclear. This study aimed to evaluate the association between sleep debt and mental well-being in school-aged children and adolescents. **Methods:** This cross-sectional study included 4967 children and adolescents aged 9–18 years from eight provincial-level regions in China, using data from a multicenter school-based survey conducted within the framework of the Chinese National Survey on Students’ Constitution and Health (CNSSCH). Participants self-reported relevant sleep parameters, and the sleep debt was calculated based on the difference in sleep duration between weekdays and weekends. The main outcome was mental well-being. Adjusted covariates were age, sex, urban–rural residence, body mass index (BMI), vital capacity, and waist-to-hip ratio. **Results:** A total of 4967 children and adolescents were included (mean [SD] age 12.77 [2.59] years; 2475 [49.83%] girls) with 3176 participants classified as having higher mental well-being and 1791 participants classified as having lower mental well-being. After adjustment for covariates, sleep debt was associated with greater odds of lower mental well-being (adjusted odds ratio [aOR] = 1.095; 95% CI, 1.050–1.143; *p* < 0.001). Although statistically significant, the magnitude of this association was modest. In stratified analyses, each quartile increase in sleep debt was associated with 17.8% higher odds of lower mental well-being in boys (95% CI, 8.6–27.8%), 33.8% higher odds in girls (95% CI, 23.8–44.6%), 25.3% higher odds in urban students (95% CI, 16.3–35.0%), and 28.2% higher odds in rural students (95% CI, 17.6–39.7%) (all *p* < 0.001). In addition, weekend sleep patterns significantly interacted with the sleep debt–mental well-being association (*p* for interaction < 0.05), while age-specific nonlinear patterns involving nap duration were observed among adolescents aged 15–17 years (*p* for nonlinearity = 0.004). **Conclusions:** Sleep debt showed a statistically significant but modest cross-sectional association with lower mental well-being. Numerically larger subgroup-specific estimates were observed among girls and rural students. Given the cross-sectional design, these findings should not be interpreted as evidence of causality. Despite its modest magnitude, the association may have public health relevance at the population level. The observed patterns involving weekend sleep and age-specific nap duration warrant further evaluation in longitudinal and, where appropriate, intervention studies.

## 1. Introduction

The Lancet Global Adolescent Health Report 2nd Edition warns that adolescent well-being has reached the critical stage, with mental well-being crises increasing at unprecedented rates; projections indicate that more than 1 billion adolescents worldwide will suffer severe health deficits by 2030 under existing interventions [1]. In recent years, the incidence rates of common mental disorders (i.e., depression and anxiety) have continued to increase sharply among the younger generation [2]. As of 2021, 252 million young people have mental disorders worldwide (9.5% of all children and adolescents), and in China, more than 30 million children and adolescents suffer from mental disorders [3]. Previous studies have offered in-depth insights into mental health issues among Chinese children and adolescents [4,5]. Concerns regarding their mental well-being urgently need to be addressed as a global public health priority.

Sleep is one of the important physiological activities of children and adolescents, and adequate sleep duration and good sleep quality are essential for maintaining cognitive, emotional, and psychological development [6]. Extensive research evidence suggests that sleep deprivation and rhythm disorders are significant risk factors for psychological problems such as anxiety and depression in school-aged children and adolescents [7,8,9]. Long-term sleep deprivation has been reported to be associated with increased negative affect (NA) and decreased positive affect (PA) in adolescents [10]. However, individual sleep–wake patterns are not always constant, and weekday-to-weekend sleep differences are common among school-aged children and adolescents. “Sleep debt” generally refers to cumulative sleep deprivation resulting from insufficient sleep on school days. Operationally, it is quantified as the gap between sleep duration on free days and school days in this study. Notably, longer weekend sleep only serves as a marker of weekday sleep restriction, rather than a direct physiological measurement of sleep debt itself. Accumulation of sleep debt usually results in longer and deeper sleep in subsequent periods. Although the American Academy of Sleep Medicine (AASM) has issued age-specific recommendations for sleep duration to promote optimal health [11], the prevalence and duration of sleep debt among school-aged children and adolescents continue to rise. In the United States, more than 50% of children are reported to experience sleep debt [12]. Similarly, the 2019 White Paper on the Sleep Index of Chinese Children and Adolescents indicated that students generally exhibited poor sleep status, with widespread catch-up sleep on weekends and over half of the students failing to meet the recommended sleep duration standards [13]. This phenomenon can be attributed to various ecological and psychosocial factors, including heavy academic demands, excessive participation in extracurricular and social activities, earlier wake-up times, and delayed bedtime [14,15]. The parameters used to report sleep vary depending on the specific sleep-related problem being investigated, such as sleep duration, sleep quality, and sleep behavior [16]. In Chinese students, the negative associations between poor sleep quality, short sleep duration, and mental well-being have been well documented [17,18]. However, few studies have explored the impact of circadian parameters, such as sleep debt and social jetlag. In addition, school-aged children and adolescents at different ages show significant differences in circadian rhythm and sleep needs [19], and these differences may be further moderated by factors such as napping habits, sex, and urban–rural background.

Therefore, in this study, we analyzed cross-sectional data from children and adolescents in eight provinces in China, and after controlling for various key covariates such as age, sex, urban–rural residence, waist-to-hip ratio (WHR), and body mass index (BMI), we studied the association between sleep debt and mental well-being of children and adolescents. We hypothesized that sleep debt would be negatively linked to mental well-being, and sleep-related behaviors such as napping may produce effect modification on this association. Based on previous evidence [20], we examined cross-sectional effect modification by nap duration on the link between sleep debt and mental well-being among school-aged children and adolescents, as well as potential differences across age subgroups.

## 2. Materials and Methods

### 2.1. Study Design, Setting, and Participants

In this multicenter observational cross-sectional study, data were obtained from a prospective follow-up investigation on the impact of COVID-19 on visual acuity among students aged 6–18 years. The study was conducted across nine provinces in China (Henan, Shanxi, Gansu, Hunan, Chongqing, Guangxi, Fujian, Shanghai, and Jiangsu) from November 2019 to November 2020. The sampling framework was established based on the Chinese National Survey on Students Constitution and Health (CNSSCH), a nationwide epidemiological survey system. Detailed methodological protocols and study design for the CNSSCH program have previously been described in the literature [21]. We collected and longitudinally tracked school records, individual demographics, anthropometric measurements, sleep patterns, and psychological status in a cohort of 7545 school-aged children and adolescents. After excluding 2578 participants with incomplete school records, missing anthropometric data, or aberrant measurements of relevant covariates, the final analysis sample consisted of 4967 participants aged 9–18 years from eight provincial-level regions in China, with Jiangsu Province excluded from the final analysis. The detailed flowchart is shown in Supplementary Figure S1. Prior to initiation of the study, written informed consent was obtained from primary caregivers alongside participant consent from school-aged children and adolescents. The study protocol was approved by the Biomedical Ethics Committee of Peking University (Approval No. IRB00001052-21001) and strictly adheres to the ethical principles established in the 1964 Helsinki Declaration and subsequent amendments.

### 2.2. Assessment of Mental Well-Being

All students were administered the 14-item Warwick-Edinburgh Mental Well-being Scale (WEM-WBS) questionnaire. Each item is rated on a 5-point Likert scale, ranging from 1 (“None of the Time”) to 5 (“All of the Time”). The scale assesses different dimensions of hedonic and eudaimonic well-being, with all items phrased positively. The total score is calculated by summing all item scores, yielding a range of 14 to 70, where higher scores indicate more positive mental well-being [22]. Mental well-being was classified based on WEMWBS total scores using the established cutoff: scores > 50 indicated positive mental well-being, while scores ≤ 50 reflected negative mental well-being [23]. The Chinese version of WEM-WBS has been shown to be highly reliable and effective in assessing the mental well-being of Chinese adolescents [24].

### 2.3. Sleep Outcomes Ascertainment

Sleep duration data were gathered via standardized questionnaires, where participants self-reported their typical bedtimes and wake-up times for both weekdays and weekends. Questions included: “What time do you usually go to bed on weekdays (or weekends)?” and “What time do you usually wake up on weekdays (or weekends)?” Participants also reported daily nap durations. Mean sleep duration, in hours, was calculated separately for workdays and free days. Sleep debt was determined using the difference score (Δ = mean free-day sleep duration—mean workday sleep duration), a simplified assessment formula proposed by Professor Yonggui Yuan’s research team at Zhongda Hospital, Southeast University, with established thresholds: Δ ≤ 2 h indicates no significant sleep debt; Δ = 3 h reflects an accrued sleep debt of 1 h; Δ = 4 h indicates an accrued sleep debt of 2 h [25,26,27].

### 2.4. Covariates and Other Variables

Demographic and anthropometric data were collected using standardized protocols. Height and weight were measured by trained researchers using calibrated instruments, with standing height recorded to the nearest 0.1 cm and body weight to the nearest 0.1 kg, following WHO guidelines. BMI was calculated as weight (kg) divided by height (m)^2^. Waist and hip circumferences were measured in duplicate using a standardized protocol, and the WHR was calculated. Visual acuity was assessed under uncorrected conditions using a standard logarithmic visual acuity chart. Blood pressure was measured using a calibrated electronic sphygmomanometer, with three consecutive readings taken after 5 min of seated rest, and the mean value recorded as the final result. The vital capacity of participants was assessed using a calibrated electronic spirometer with pre-test zero calibration. Subjects performed maximal inspiration followed by complete expiration while standing, and the highest of two measurements taken within a 15 s interval was recorded as the vital capacity value.

### 2.5. Statistical Analysis

Continuous variables were expressed as mean ± standard deviation (SD) or median (interquartile range, IQR), while categorical variables were represented as frequency (percentage). For comparisons across multiple groups, one-way ANOVA followed by Tukey’s honestly significant difference (HSD) post hoc tests was used to manage the family-wise error rate. Multivariable logistic regression analyzed the associations between sleep parameters, including sleep debt, and mental well-being in school-aged children and adolescents, stratified by sex and urban–rural residence. Results were presented as odds ratios (ORs) with 95% confidence intervals (CIs). Covariates included age, sex, urban–rural classification, BMI, vital capacity, and WHR.

Participants were divided into quartiles according to sleep debt levels, and trend tests utilized median sleep debt values for each quartile. Restricted cubic spline (RCS) regression with four knots was employed to evaluate potential nonlinear relationships between sleep debt and mental well-being. Subgroup and interaction analyses were conducted to determine if these associations were influenced by other sleep-related parameters.

We conducted two sensitivity analyses. First, we performed stratified analyses based on age, WHR, vital capacity, uncorrected visual acuity, BMI, SBP, and DBP, adjusting for confounders, and compared these results with our current findings. Second, we addressed data from a subset of samples with unusual sleep patterns (e.g., staying up late, severe insomnia) by excluding those meeting any of the following criteria: sleep duration on weekdays/weekends < 4 h or >12 h; wake-up time on weekdays/weekends < 4:00 a.m. or >12:00 p.m.; bedtime on weekdays/weekends < 8:00 p.m. or >12:00 a.m. We then conducted logistic regression analyses stratified by age, sex, and urban–rural residence to further explore associations.

All statistical analyses were performed using R version 4.5.0, with a two-tailed α level set at 0.05 for significance determination.

## 3. Results

### 3.1. Baseline Characteristics

In this cross-sectional study of 4967 school-aged children and adolescents (3176 with higher mental well-being status and 1791 with lower mental well-being), the latter group was characterized by older age, higher BMI, greater vital capacity, poorer visual acuity, elevated blood pressure, and a higher proportion of girls and rural residents. They also exhibited adverse sleep patterns, including later weekday bedtimes, earlier wake-up times, shorter sleep durations on both weekdays and weekends, and greater sleep debt compared to their higher mental well-being peers (all *p* < 0.05; see Table 1).

### 3.2. Geographic Distribution Across Provinces

**Figure 1:**
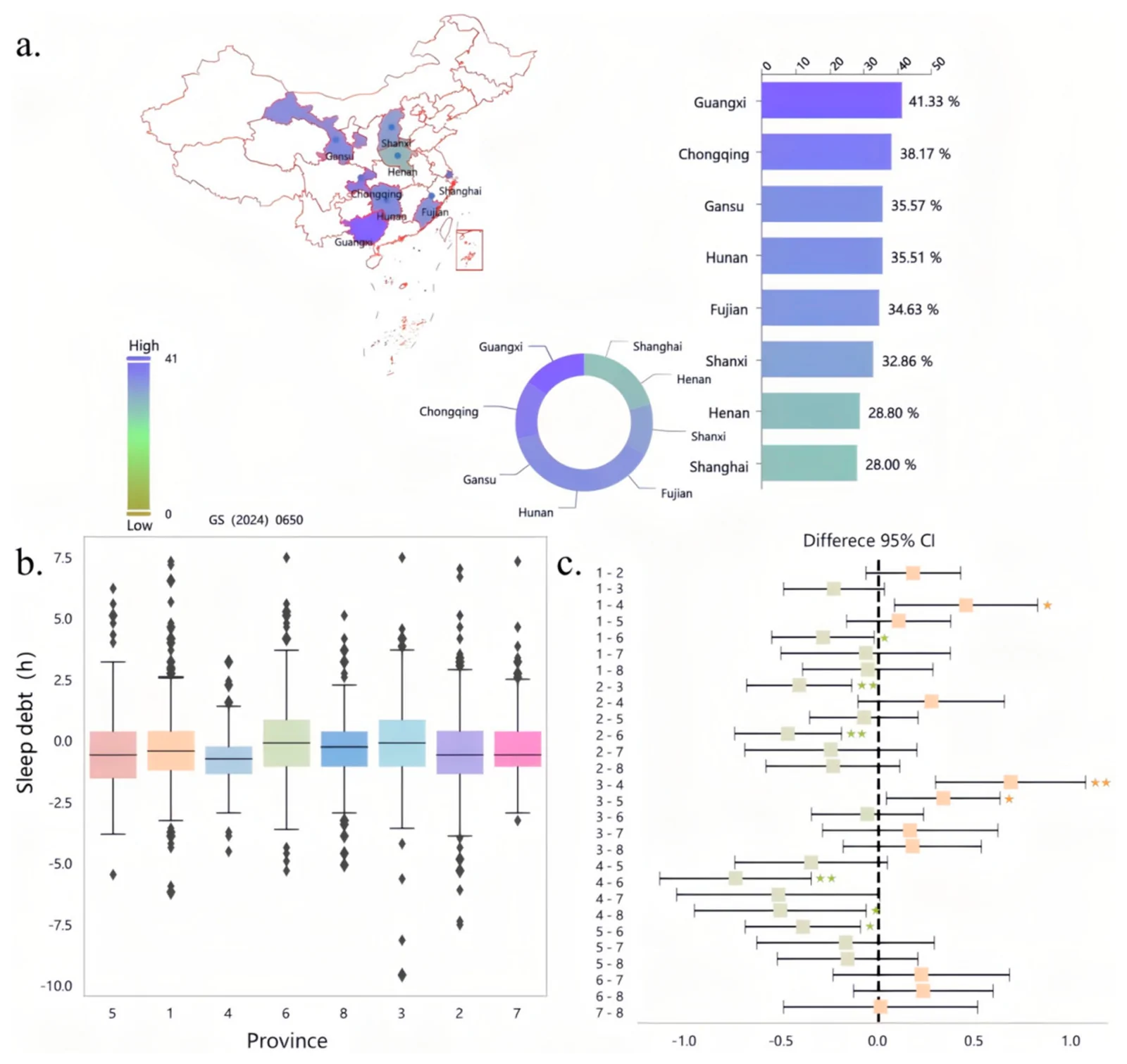
Provincial distribution of lower mental well-being in Chinese students. (**a**) Geographic distribution of children and adolescents with lower mental well-being across provinces; (**b**) Geographic distribution of children and adolescents with sleep debt across provinces; (**c**) Interprovincial comparison of sleep debt burden among children and adolescents. The color gradient in (**a**) represents the province-specific prevalence of lower mental well-being, with purple indicating higher prevalence and green indicating lower prevalence. The different box colors in (**b**) distinguish the provinces, and black diamond symbols indicate outliers. In (**c**), squares represent the estimated pairwise mean differences, and horizontal lines represent 95% confidence intervals; green and orange squares indicate negative and positive mean differences, respectively, and the vertical dashed line indicates zero difference. Asterisks indicate statistically significant Tukey-adjusted pairwise comparisons, with the asterisk colors corresponding to the direction of the mean difference (green, negative; orange, positive): * *p* < 0.05; ** *p* < 0.001. Notes: 1, Guangxi; 2, Chongqing; 3, Gansu; 4, Hunan; 5, Fujian; 6, Shanxi; 7, Henan; 8, Shanghai.

As shown in Figure 1, there were significant differences in the distribution of participants with lower mental well-being and sleep debt across eight Chinese provinces. The analysis revealed significantly higher prevalence of lower mental well-being in Guangxi (41.33%), Chongqing (38.17%), and Gansu (35.57%) (Figure 1a). Notably, the heaviest burden of sleep debt in school-aged children and adolescents was observed in Shanxi and Gansu provinces (Figure 1b). These geographic patterns suggest that there are potential regional influences on psychological well-being and sleep behaviors. Post hoc pairwise comparisons with Tukey’s adjustment revealed significant differences between multiple groups (Figure 1c). Most notably, Hunan showed consistently higher values than other groups, with significant differences compared to Guangxi (mean difference = 0.452, 95% CI [0.082, 0.822], *p* = 0.004), Gansu (0.683 [0.295, 1.071], *p* < 0.001), and Shanxi (−0.74 [−1.131, −0.349], *p* < 0.001). Chongqing showed significantly lower values than Gansu (−0.410 [−0.682, −0.139], *p* < 0.001) and Shanxi (−0.468 [−0.744, −0.191], *p* < 0.001). The most significant contrast was observed between Gansu and Hunan (difference = 0.683), while Fujian, Henan, and Shanghai generally showed no significant differences with most other groups (all *p* > 0.05 after adjustment).

### 3.3. Sleep-Related Predictors for Mental Well-Being Outcomes

**Figure 2:**
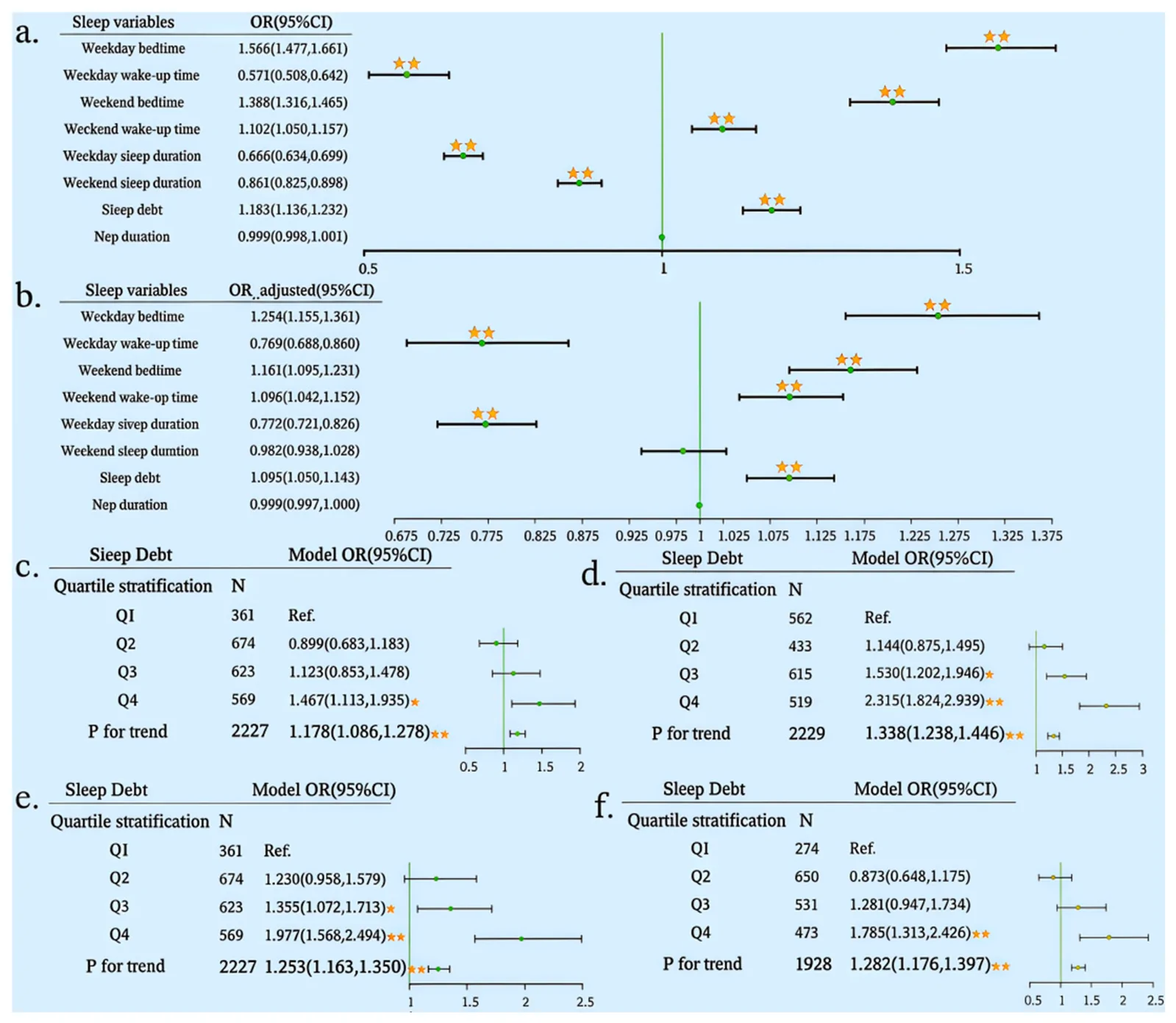
Association of sleep-related parameters and sleep debt with students’ mental well-being. (**a**) Unadjusted models of relevant sleep parameters; (**b**) adjusted models of relevant sleep parameters; (**c**) logistic regression model of sleep debt in boys; (**d**) logistic regression model of sleep debt in girls; (**e**) logistic regression model of sleep debt in urban students; (**f**) logistic regression model of sleep debt in rural students. Adjusted covariates: Age, sex, urban–rural residence, BMI, vital capacity, WHR. Notes: * *p* < 0.05; ** *p* < 0.01.

Figure 2 shows significant associations between sleep patterns and mental well-being in school-aged children and adolescents. Logistic regression analyses demonstrated that multiple sleep parameters were significantly associated with lower mental well-being in children and adolescents (Figure 2a, all *p* < 0.001). After adjusting for age, sex, urban–rural classification, BMI, vital capacity, and waist-to-hip ratio, we found that these associations remained significant (Figure 2b, all *p* < 0.001): weekday bedtime (adjusted OR [95% CI]: 1.254 [1.155–1.361]), weekday wake-up time (0.769 [0.688–0.860]), weekend bedtime (1.161 [1.095–1.231]), weekend wake-up time (1.096 [1.042–1.152]), weekday sleep duration (0.772 [0.721–0.826]), and sleep debt (1.095 [1.050–1.143]). Furthermore, we examined the trend regression associations between sleep debt and mental well-being among school-aged children and adolescents stratified by sex and urban–rural residence. Our analyses revealed that each quartile increase in sleep debt was associated with significantly lower mental well-being: 17.8% (95% CI, 8.6–27.8%) in boys (Figure 2c), 33.8% (23.8–44.6%) in girls (Figure 2d), 25.3% (16.3–35.0%) in urban students (Figure 2e), and 28.2% (17.6–39.7%) in rural students (Figure 2f), all *p* < 0.001.

### 3.4. Subgroup Analysis of the Association Between Sleep Debt and Mental Well-Being

**Figure 3:**
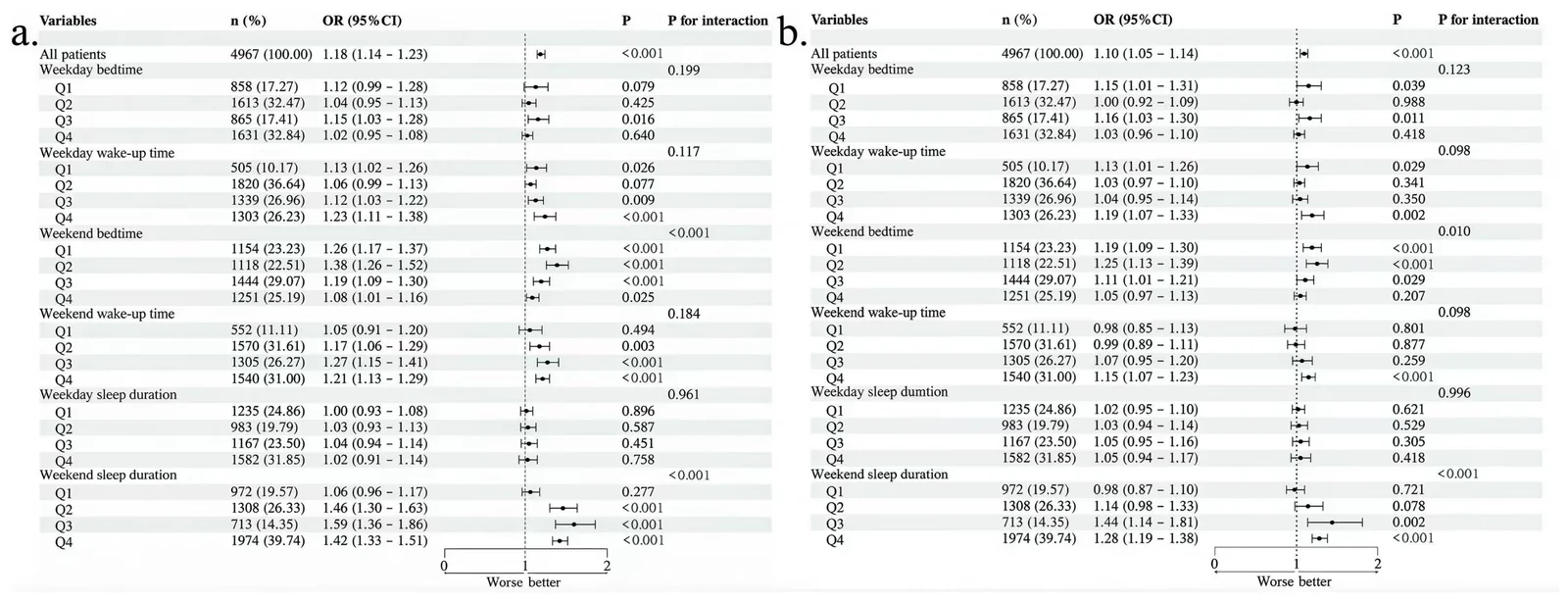
Subgroup analysis of mental well-being-associated sleep parameters. (**a**) Unadjusted model; (**b**) Adjusted models of relevant sleep parameters. Covariates: age, sex, urban–rural residence, WHR, BMI, and vital capacity.

Figure 3 presents the subgroup analysis of the link between sleep debt and mental well-being, highlighting significant effect modifications by weekend bedtime and sleep duration (*p* for interaction < 0.001 for both, Figure 3a). After covariate adjustment, the highest lower mental well-being was noted in subgroups with a Q2 weekend bedtime (OR = 1.25, 95% CI, 1.13–1.39, *p* for interaction = 0.010) and a Q3 weekend sleep duration (OR = 1.44, 95% CI, 1.14–1.81, *p* for interaction < 0.001, Figure 3b). We found that greater sleep debt and adverse weekend sleep patterns were linked to progressively worse mental well-being, indicating a synergistic interaction between these factors.

### 3.5. Age-Stratified Nonlinear Association Patterns for Nap Duration

**Figure 4:**
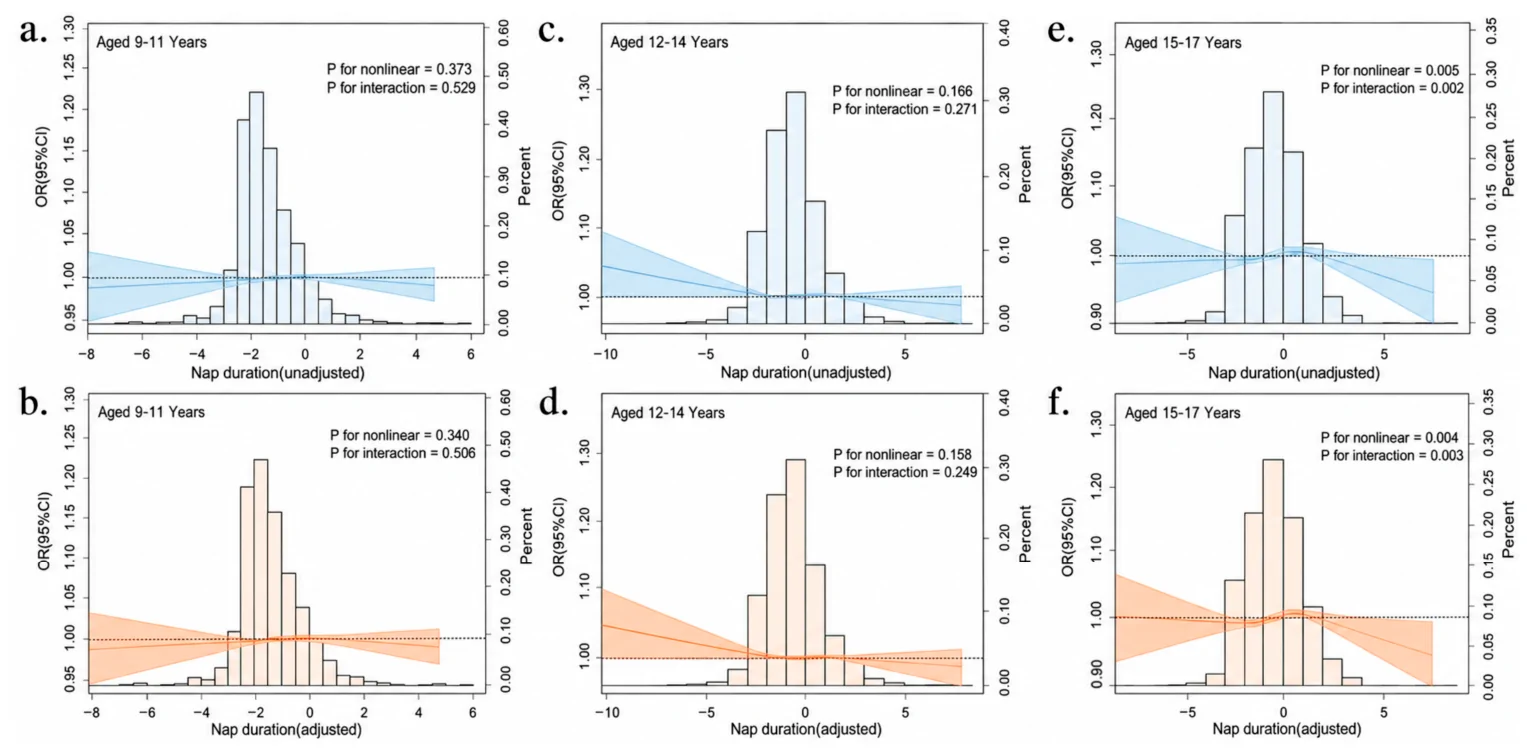
Age-stratified nonlinear interaction analysis using RCS models. (**a**,**b**) Aged 9–11 years; (**c**,**d**) Aged 12–14 years; (**e**,**f**) Aged 15–17 years.

Figure 4 illustrates age-specific trends in the association between nap duration and mental well-being with increasing sleep debt. After adjusting for sex, urban–rural classification, WHR, and BMI, significant interactions between sleep debt and nap duration on lower mental well-being were found only in adolescents aged 15–17 years (*p* for nonlinearity < 0.05, *p* for interaction < 0.05; Figure 4e,f). No such interactions were observed in children aged 9–11 or 12–14 years (all *p* for interaction > 0.05; Figure 4a–d).

### 3.6. Age-Related Differences in Sleep Patterns

**Figure 5:**
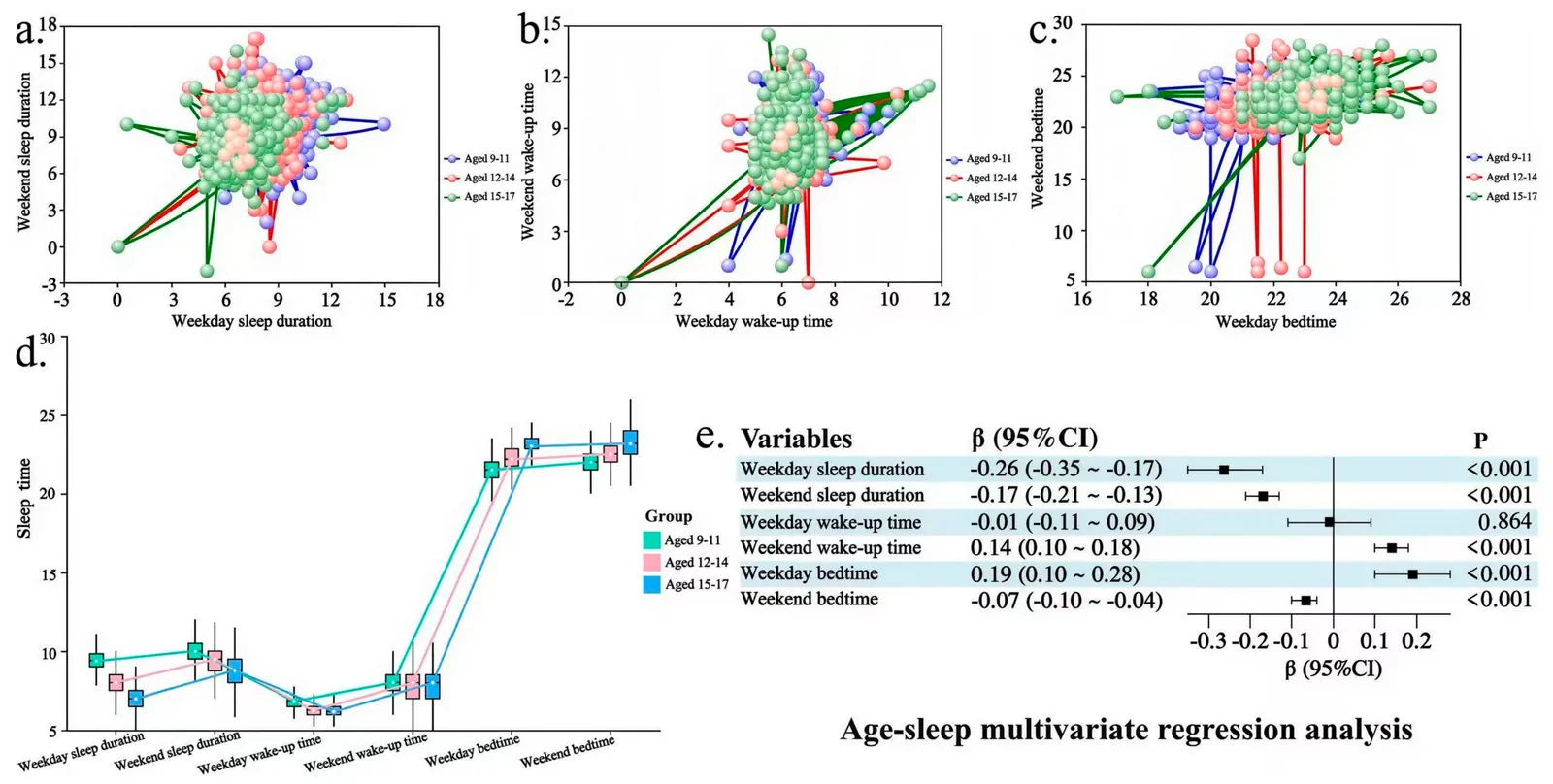
Age-stratified distribution characteristics of sleep parameters and the association between age and sleep parameters. (**a**) Sleep duration on weekdays/weekends; (**b**) Wake-up time on weekdays/weekends; (**c**) Bedtime on weekdays/weekends; (**d**) Age-Stratified Comparison of sleep parameter distributions; (**e**) Association between age and sleep parameters.

Figure 5 illustrates the age-stratified relationships between sleep parameters and nap-induced physiological responses, highlighting age’s moderating role in sleep-nap dynamics. Sleep duration (Figure 5a) and wake-up times (Figure 5b) on school days and weekends show a clear age-related trend: adolescents aged 15–17 years exhibit shorter sleep durations and earlier wake-up times than those aged 12–14 and 9–11. Bedtime patterns (Figure 5c) display an inverse trend, with the youngest group retiring earliest, followed by the middle and older groups. These differences are further corroborated by a comprehensive comparison of sleep parameters across age groups (Figure 5d), revealing a consistent trend. Significant linear associations were found between age and key sleep variables. Age negatively correlates with sleep duration on weekdays (β = −0.26, 95% CI, −0.35 to −0.17) and weekends (β = −0.17, 95% CI, −0.21 to −0.13) (Figure 5e). Conversely, age positively correlates with weekend wake-up times (β = 0.14, 95% CI, 0.10 to 0.18) and weekday bedtime (β = 0.19, 95% CI, 0.10 to 0.28), while negatively correlating with weekend bedtime (β = −0.07, 95% CI, −0.10 to −0.04). No significant association was observed between age and weekday wake-up time (*p* > 0.05).

### 3.7. Sensitivity Analyses

After adjusting for sex and urban–rural residence, stratified analyses by age, waist-to-hip ratio, vital capacity, visual acuity, BMI, SBP, and DBP consistently showed significant associations between sleep debt and mental well-being in school-aged children and adolescents (Figure S2, Supplementary Materials). These associations remained robust after excluding participants with abnormal sleep patterns (e.g., staying up late and insomnia) and adjusting for potential confounders across age, sex, and urban–rural residence strata (Figure S3, Supplementary Materials).

## 4. Discussion

This study is, to our knowledge, the first multi-province study to systematically characterize the graded, quartile-based cross-sectional association between sleep debt and lower mental well-being among Chinese school-aged children and adolescents. By integrating sleep debt quartiles, weekend sleep patterns, age-specific nap duration, and subgroup analyses, this study provides novel population-based evidence that sleep debt may serve as a modest but potentially relevant behavioral marker of lower mental well-being in this population. The key findings were as follows: (1) each quartile increase in sleep debt was associated with a 9.5% higher odds of lower mental well-being (95% CI, 5.0–14.3%), with the sex-stratified estimate numerically larger in girls than in boys and the residence-stratified estimate slightly larger in rural than in urban students; (2) weekday-to-weekend sleep differences were associated with mental well-being, but should be interpreted as markers of irregular sleep patterns rather than evidence of physiological sleep compensation; and (3) nap duration showed age-specific associations with mental well-being among adolescents aged 15–17 years. These findings highlight potentially relevant subgroup patterns and sleep-related behavioral markers that warrant further investigation in longitudinal and intervention studies.

In this study, the average weekday sleep duration for Chinese school-aged children and adolescents was 8.12 ± 1.31 h, notably exceeding that of U.S. students in grades 9–12 (7 h 12 min) and French students aged 15–19 (7 h 45 min) [28]. Despite the older age groups in these international comparisons potentially having different sleep needs, the findings align with the recommended 8–10 h per 24 h for this demographic [29]. Previous research among Chinese students has consistently documented disruptions in sleep rhythms, including insufficient sleep duration during school days and excessive compensatory sleep on weekends, which are associated with increased risks to adolescent mental well-being [30,31]. The current study’s findings further corroborate the significant association between disrupted sleep patterns and mental well-being in school-aged children and adolescents. However, these earlier studies did not further investigate the specific relationship, particularly in relation to sleep debt. Huang et al. [31] proposed an educational stage stratification method to analyze the links between adolescent social jetlag, nap duration, and mental well-being. This approach offers a valuable framework for age-stratified interaction analysis of nap-related factors in our research. The findings highlight the critical importance of maintaining a stable circadian rhythm, characterized by consistent bedtimes and sufficient sleep, to reduce sleep debt and safeguard the mental well-being of school-aged children and adolescents. Our study found a significant correlation between sleep patterns and mental well-being in school-aged children and adolescents. After adjusting for potential confounders, delayed bedtimes on both weekdays and weekends, earlier wake-up times, and reduced sleep durations were independently associated with lower mental well-being (all *p* values < 0.001). Importantly, a statistically significant association was identified: each quartile increase in sleep debt corresponded to a 9.5% increase in the odds of lower mental well-being (95% confidence interval: 5.0–14.3%). Although this association was statistically significant, its magnitude was modest and should not be interpreted as indicating a large individual-level clinical effect. Rather, the practical relevance of this finding lies mainly in its potential population-level implications, given the high prevalence of sleep insufficiency and irregular sleep patterns among school-aged children and adolescents. Therefore, sleep debt should be considered a modest but potentially meaningful behavioral marker of lower mental well-being, rather than a dominant or standalone determinant. This finding aligns with Shen et al. [32], who showed that cumulative sleep debt predicts heightened negative mood and diminished low-arousal positive affect (low arousal PA) the next day. Like sleep debt, social jetlag serves as a mediator between circadian preference and mental well-being, often indicating accumulated sleep debt [33,34]. Studies on Chinese school-aged children and adolescents have consistently demonstrated a significant positive correlation between social jetlag and lower mental well-being [31,35,36], reinforcing our study’s conclusions.

Associations remained statistically significant within each sex and residence stratum. Notably, the sex-stratified estimate was numerically larger in girls than in boys, with 33.8% higher odds of lower mental well-being per quartile increment in sleep debt (95% CI, 23.8–44.6%), while the residence-stratified estimate was slightly larger in rural than in urban students, with 28.2% higher odds (95% CI, 17.6–39.7%). Additionally, a higher percentage of females have been reported to experience at least one hour of sleep debt (58.8% vs. 47.5% for males) and poor sleep quality (11.1% vs. 8.4% for males) [37]. These numerical subgroup differences should be regarded as descriptive patterns rather than evidence of greater susceptibility. These descriptive subgroup patterns, together with the age-specific nap-related finding observed among adolescents aged 15–17 years, may reflect a combination of developmental, psychosocial, and contextual factors. The numerically larger estimate among girls may be related to sex-specific differences in pubertal development, emotional regulation, perceived academic pressure, and sleep quality. Adolescents aged 15–17 years may experience greater circadian delay, heavier academic demands, and more pronounced weekday sleep restriction, which may contribute to the age-specific nap-related pattern observed in this group. For rural students, differences in family socioeconomic resources, educational environments, access to health and educational resources, and sleep-supportive conditions may be relevant to the slightly larger estimate observed in this subgroup. However, these subgroup findings should be interpreted cautiously. Stratified estimates alone do not establish statistically significant effect modification. Because this study was cross-sectional and did not include detailed biological, socioeconomic, or environmental measurements, these explanations remain tentative and should be tested in future longitudinal studies. The provincial distribution analysis showed relatively higher proportions of lower mental well-being in Guangxi, Chongqing, and Gansu, and a relatively heavier sleep debt burden in Shanxi and Gansu. However, these provincial patterns should be interpreted as descriptive sample characteristics rather than evidence of regional causal mechanisms. Although previous studies have suggested that socioeconomic disadvantage and contextual inequalities may be associated with poorer sleep quality and shorter sleep duration in children and adolescents [38,39,40,41], the present study did not collect province-level socioeconomic, educational, family, or health-resource indicators. Therefore, the cross-sectional design only allows descriptive observation of provincial differences and does not permit causal inference regarding the contextual factors underlying these regional patterns. Future studies incorporating multilevel regional, socioeconomic, educational, and family-level variables are needed to clarify whether and how contextual factors contribute to regional disparities in sleep debt and mental well-being.

Sleep debt, a state of disrupted sleep homeostasis, shows a significant association with poorer mental well-being among school-aged children and adolescents. While the existence of this issue is widely recognized, the underlying neuropathological mechanisms remain to be fully elucidated. School-aged children and adolescents often face sleep deprivation, leading to the accumulation of sleep debt. A recent report has provided a possible explanation for the neuropathological mechanisms behind sleep deprivation, highlighting the role of TOP1-mediated microglial activation in hippocampal neuronal damage and the resulting neuroinflammatory response [42]. A study suggests that the cortex-basal ganglia may mediate the effects of sleep deficiency on depression, cognitive issues, and crystallized intelligence [43]. Complex underlying mechanisms underpin the cross-sectional association between sleep debt and mental well-being in school-aged children and adolescents. Theoretical models, such as the role of slow-wave sleep in regulating positive emotions and rapid eye movement sleep in processing emotional experiences, offer insights into these mechanisms [44]. Admittedly, these mechanisms are merely speculative. As an observational study without physiological test data, the current research cannot directly verify such neurobiological hypotheses, and further targeted studies are required to explore the underlying mechanisms.

Weekend sleep patterns may reflect weekday sleep restriction and irregular sleep schedules among school-aged children and adolescents. However, because this study was cross-sectional, longer weekend sleep should be interpreted as a behavioral marker of weekday sleep insufficiency rather than evidence of physiological compensation. Previous evidence has suggested that excessive weekend sleep catch-up (>120 min) may be associated with lower self-reported well-being among adolescents with insufficient sleep (<7 h) [45]. In our subgroup analysis, weekend sleep patterns modified the association between sleep debt and mental well-being. Longer weekend sleep was associated with less severe lower mental well-being related to weekday sleep debt in one subgroup (Q1: OR = 0.98, 95% CI: 0.87–1.10), whereas later weekend bedtimes (Q2: OR = 1.25, 95% CI, 1.13–1.39) and longer weekend sleep durations (Q3: OR = 1.44, 95% CI, 1.14–1.81) were associated with lower mental well-being after covariate adjustment. Academic stress has also been associated with adolescent mental well-being, particularly among Chinese high school students [46]. Therefore, weekend sleep timing and duration may capture complex behavioral patterns related to school-day sleep restriction, academic demands, and irregular rest schedules. These findings suggest that weekend sleep patterns are important sleep-related markers associated with lower mental well-being, but they do not establish whether weekend sleep changes compensate for weekday sleep debt or directly influence mental well-being. Future longitudinal studies using objective sleep measurements are needed to clarify the temporal and causal relationships among weekday sleep restriction, weekend sleep patterns, and mental well-being.

Age, night sleep duration, and daytime nap duration are primary determinants of circadian rhythm type, known as social jetlag, with relative feature importances in a random forest model of 0.441, 0.349, and 0.204, respectively [47]. In China, school-aged children and adolescents are influenced by a cultural emphasis on napping, resulting in lower social jetlag compared to European populations. Our study found that adolescents aged 15–17 years exhibited poorer sleep quality, higher sleep debt-to-lower mental well-being ratios, and more sensitive nap-related adjustments than those aged 9–14, likely due to puberty-related factors and increased academic pressure. Specifically, adolescents aged 15–17 years slept less, woke earlier on both school days and weekends, and went to bed later, indicating prevalent sleep deprivation in this age group. Further analysis revealed significant positive correlations between age and certain sleep parameters: weekday bedtime (β = 0.19, 95% CI, 0.10–0.28) and weekend wake time (β = 0.14, 95% CI, 0.10–0.18). These findings highlight the sleep patterns of older adolescents (15–17 years), who tend to sleep late on weekdays due to academic pressures and wake late on weekends following a demanding week, underscoring their severe sleep deprivation. Age-appropriate nap patterns may be associated with differences in mental well-being in this age group, although this observational finding requires confirmation in longitudinal or intervention studies. Notably, no significant link was found between age and weekday wake-up time, likely due to uniform school start times across age groups. The age-stratified association between sleep debt and mental well-being persisted after excluding specific sleep data, such as late nights and insomnia, and adjusting for confounders (OR = 1.924; 95% CI, 1.753–2.113; *p* for trend < 0.05). Additionally, a nonlinear interaction effect of naps was identified in adolescents aged 15–17 years in the study (*p* for interaction = 0.002). Zhang et al. [48] found that children initially exhibit an early-sleeping circadian rhythm, which shifts to a late-sleeping pattern during adolescence. This transition, along with increasing age and maturity, results in greater social jetlag and sleep debt. Yamada et al. [47] identified a U-shaped relationship between nap length and health risk, recommending short naps (<30 min) for optimal health benefits. Liu et al. [49] suggested that napping positively impacts the mental well-being of Chinese primary school students. These studies provide a possible background for interpreting the age-specific associations observed in our study; however, whether nap practices can improve mental well-being in adolescents aged 15–17 years should be tested in future longitudinal or intervention research.

### Strengths and Limitations

This study has several strengths. First, to our knowledge, this is the first multi-province study to systematically characterize the graded, quartile-based cross-sectional association between sleep debt and lower mental well-being among Chinese school-aged children and adolescents. Second, the study included a relatively large sample from eight provincial-level regions, which allowed us to explore potential heterogeneity by sex, age, urban–rural residence, weekend sleep patterns, and nap duration. Third, sleep debt was analyzed not only as a general sleep-related indicator but also in relation to specific sleep-pattern correlates, providing a more nuanced epidemiological perspective beyond total sleep duration alone. Fourth, standardized procedures were used to collect anthropometric and physical fitness indicators, and the multivariable models adjusted for several demographic and physiological covariates, including age, sex, urban–rural residence, BMI, WHR, and vital capacity. These strengths provide novel population-based evidence for understanding sleep-related behavioral markers associated with lower mental well-being in school-aged children and adolescents.

Despite its strengths, this study has notable limitations. Firstly, as a cross-sectional analysis, it cannot establish causal relationships or track the temporal dynamics between cumulative sleep debt and lower mental well-being in school-aged children and adolescents. Longitudinal studies with repeated measures are needed to clarify the underlying mechanisms. Secondly, restricting the study to individuals aged 9–18 may limit the generalizability of the findings to younger pediatric populations. Thirdly, sleep parameters were based on retrospective subjective reports, lacking real-time sleep diaries or objective monitoring. Fourthly, although subgroup analyses were performed, some groups (e.g., sex- and urban/rural-specific quartiles) had small sample sizes, indicating a need for future surveys with targeted sampling strategies. In addition, this simple sleep duration difference metric is unable to precisely calculate the total amount of long-term accumulated sleep debt. It does not account for individual daily physiological sleep needs, nor does it capture sustained sleep insufficiency over consecutive days. To address these shortcomings, future studies will adopt more objective and standardized assessment indicators to quantitatively measure sleep debt, which can achieve a more accurate and comprehensive evaluation of long-term sleep insufficiency status. Finally, residual confounding from unmeasured variables cannot be excluded. Although the adjusted models included age, sex, urban–rural residence, BMI, vital capacity, and WHR, several important factors that may influence both sleep patterns and mental well-being were unavailable in the present dataset, including socioeconomic status, academic stress, physical activity [50], screen time [51], and prior mental health conditions. The absence of these covariates limits our ability to draw causal interpretations, and the observed associations should not be attributed solely to sleep debt. Future longitudinal studies should incorporate these behavioral, socioeconomic, academic, and psychological variables to provide more robust evidence. Notably, although the magnitude of the observed association was modest, sleep debt is common among school-aged children and adolescents. Therefore, even a modest association may have meaningful population-level relevance because it could correspond to a substantial absolute number of affected individuals. In addition, numerous stratified and interaction analyses were performed without multiple testing correction, raising the risk of Type I error. All subgroup findings are preliminary and warrant replication.

## 5. Conclusions

To our knowledge, this is the first multi-province study to systematically characterize the graded, quartile-based cross-sectional association between sleep debt and lower mental well-being among Chinese school-aged children and adolescents. In the overall adjusted model, a one-quartile increase in sleep debt was associated with 9.5% higher odds of lower mental well-being (aOR = 1.095; 95% CI, 1.050–1.143). Numerically larger subgroup-specific estimates were observed among girls and rural students. Although statistically significant, the magnitude of the overall association was modest and should not be interpreted as evidence of causality. Its potential public health relevance lies primarily at the population level, given the common occurrence of insufficient and irregular sleep in this population. The observed patterns involving weekday-to-weekend sleep differences and age-specific nap duration should be regarded as hypothesis-generating and warrant further evaluation in longitudinal and, where appropriate, intervention studies.

## Figures and Tables

**Table 1:** Baseline characteristics of students with mental well-being.

Variables	Total (n = 4967)	Higher Mental Well-Being Group (n = 3176)	Lower Mental Well-Being Group (n = 1791)	*p*
Age (years), Mean ± SD	12.77 ± 2.59	12.29 ± 2.57	13.60 ± 2.43	<0.001
Sex, n (%)				<0.001
Boys	2492 (50.17)	1657 (52.17)	835 (46.62)	
Girls	2475 (49.83)	1519 (47.83)	956 (53.38)	
Urban–rural residence, n (%)				0.043
Urban	2754 (55.45)	1795 (56.52)	959 (53.55)	
Rural	2213 (44.55)	1381 (43.48)	832 (46.45)	
Uncorrected visual acuity, right eye, Mean ± SD	4.69 ± 0.42	4.73 ± 0.40	4.63 ± 0.44	<0.001
Uncorrected visual acuity, left eye, Mean ± SD	4.72 ± 0.40	4.75 ± 0.39	4.66 ± 0.41	<0.001
Waist-to-hip ratio, Mean ± SD	0.81 ± 0.08	0.82 ± 0.08	0.80 ± 0.07	<0.001
BMI (kg/m^2^), Mean ± SD	19.46 ± 3.59	19.25 ± 3.62	19.81 ± 3.51	<0.001
SBP (mmHg), Mean ± SD	107.01 ± 12.57	106.34 ± 12.70	108.21 ± 12.23	<0.001
DBP (mmHg), Mean ± SD	65.74 ± 9.26	65.43 ± 9.28	66.29 ± 9.21	0.002
Vital capacity (mL), Mean ± SD	2419.61 ± 879.99	2362.07 ± 883.42	2521.65 ± 864.76	<0.001
Weekday bedtime (h), Mean ± SD	22.30 ± 1.05	22.13 ± 1.02	22.60 ± 1.04	<0.001
Weekday wake-up time (h), Mean ± SD	6.42 ± 0.60	6.48 ± 0.62	6.31 ± 0.56	<0.001
Weekend bedtime (h), Mean ± SD	22.53 ± 1.28	22.37 ± 1.26	22.81 ± 1.27	<0.001
Weekend wake-up time (h), Mean ± SD	7.98 ± 1.20	7.93 ± 1.13	8.07 ± 1.29	<0.001
Weekday sleep duration (h), Mean ± SD	8.12 ± 1.31	8.36 ± 1.27	7.71 ± 1.27	<0.001
Weekend sleep duration (h), Mean ± SD	9.43 ± 1.40	9.53 ± 1.32	9.24 ± 1.52	<0.001
Nap duration (min), Mean ± SD	40.77 ± 44.74	41.36 ± 41.57	39.72 ± 49.86	0.215
Sleep debt, n (%)				<0.001
No	3758 (75.66)	2526 (79.53)	1232 (68.79)	
Yes	1209 (24.34)	650 (20.47)	559 (31.21)	

## Data Availability

The data supporting the findings of this study are not publicly available due to participant privacy and ethical restrictions. De-identified data may be made available from the corresponding authors upon reasonable request and subject to approval by the relevant ethics committee and applicable data governance requirements.

## Supplementary Materials

The following supporting information can be downloaded at: https://www.mdpi.com/article/10.3390/healthcare14162503/s1. Figure S1: Flowchart of participant selection in the current study; Figure S2: Stratified associations between sleep debt and lower mental well-being. (a) Unadjusted model; (b) model adjusted for sex and urban–rural residence; Figure S3: Stratified associations between sleep debt and lower mental well-being after excluding participants with abnormal sleep parameters. (a) Age-stratified unadjusted model; (b) age-stratified model adjusted for sex, WHR, BMI, and vital capacity; (c) unadjusted subgroup model by sex and urban–rural residence; (d) model adjusted for age, WHR, BMI, and vital capacity.

## Abbreviations

The following abbreviations are used in this manuscript:

aOR	Adjusted odds ratio
AASM	American Academy of Sleep Medicine
BMI	Body mass index
CI	Confidence interval
CNSSCH	Chinese National Survey on Students’ Constitution and Health
DBP	Diastolic blood pressure
IQR	Interquartile range
OR	Odds ratio
Q1	First quartile
Q2	Second quartile
Q3	Third quartile
Q4	Fourth quartile
RCS	Restricted cubic spline
SBP	Systolic blood pressure
SD	Standard deviation
WEM-WBS	Warwick–Edinburgh Mental Well-being Scale
WHR	Waist-to-hip ratio
