# Supplementary material for: Association Between Sleep Debt and Mental Well-Being in School-Aged Children and Adolescents in China: A Multi-Province, Cross-Sectional, Observational Study
Source: Healthcare (Basel). 2026 Aug 12;14(16):2503. doi: 10.3390/healthcare14162503 (PMC13512398; doi:10.3390/healthcare14162503)
Supplement: Supplementary file 1 [file healthcare-14-02503-s001.zip › healthcare-4384079-supplementary.pdf]

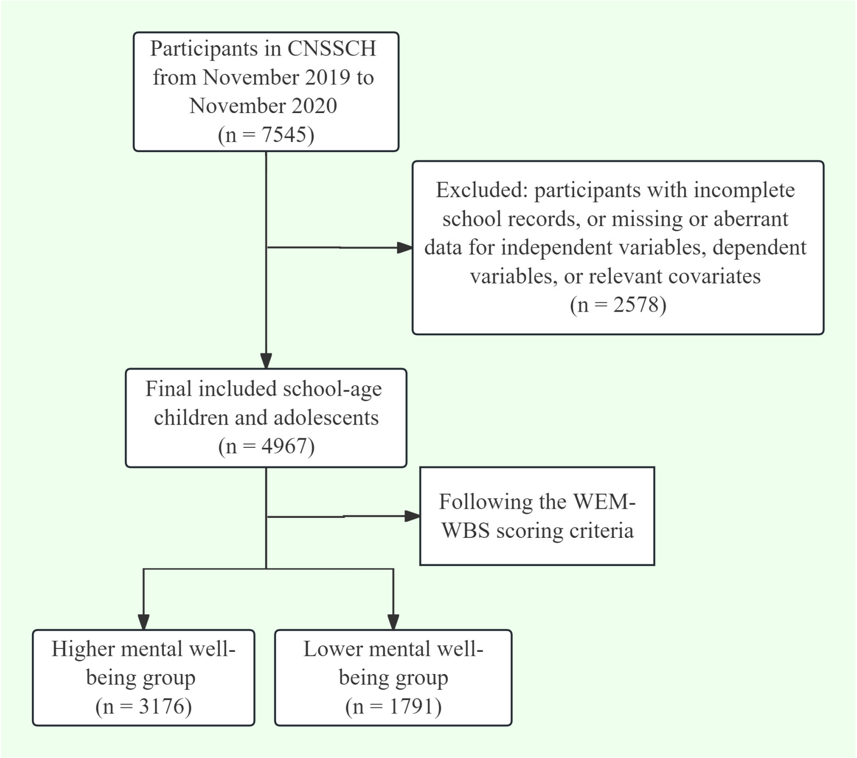

Figure S1. Flowchart of participant selection in the current study.

a.

Variables	n (%)	OR (95%CI)	P	P for interaction
All patients	4967 (100.00)	1.18 (1.14 ~ 1.23)	<.001	
Age				0.412
Q1	650 (13.09)	1.07 (0.91 ~ 1.26)	0.437	
Q2	1550 (31.21)	1.16 (1.07 ~ 1.27)	<.001	
Q3	1143 (23.01)	1.07 (1.00 ~ 1.16)	0.064	
Q4	1624 (32.70)	1.06 (0.99 ~ 1.14)	0.071	
Waist-hip ratio				0.990
Q1	1239 (24.94)	1.15 (1.06 ~ 1.24)	<.001	
Q2	1244 (25.05)	1.16 (1.07 ~ 1.26)	<.001	
Q3	1242 (25.01)	1.17 (1.08 ~ 1.27)	<.001	
Q4	1242 (25.01)	1.16 (1.07 ~ 1.27)	<.001	
Vital capacity				<.001
Q1	1240 (24.96)	1.21 (1.10 ~ 1.34)	<.001	
Q2	1243 (25.03)	1.29 (1.18 ~ 1.41)	<.001	
Q3	1241 (24.98)	1.14 (1.05 ~ 1.23)	0.001	
Q4	1243 (25.03)	1.03 (0.96 ~ 1.11)	0.346	
Right naked eye vision				0.028
Q1	1109 (22.33)	1.12 (1.03 ~ 1.22)	0.006	
Q2	1228 (24.72)	1.13 (1.04 ~ 1.23)	0.004	
Q3	695 (13.99)	1.08 (0.97 ~ 1.20)	0.180	
Q4	1935 (38.96)	1.26 (1.18 ~ 1.35)	<.001	
Left naked eye vision				0.769
Q1	1028 (20.70)	1.15 (1.06 ~ 1.26)	0.001	
Q2	1150 (23.15)	1.19 (1.09 ~ 1.30)	<.001	
Q3	778 (15.66)	1.11 (1.01 ~ 1.24)	0.039	
Q4	2011 (40.49)	1.18 (1.10 ~ 1.26)	<.001	
BMI				0.040
Q1	1242 (25.01)	1.24 (1.13 ~ 1.37)	<.001	
Q2	1241 (24.98)	1.13 (1.05 ~ 1.23)	0.002	
Q3	1242 (25.01)	1.24 (1.14 ~ 1.34)	<.001	
Q4	1242 (25.01)	1.08 (1.00 ~ 1.17)	0.041	
SBP				0.136
Q1	1216 (24.48)	1.20 (1.10 ~ 1.32)	<.001	
Q2	1114 (22.43)	1.25 (1.15 ~ 1.37)	<.001	
Q3	1339 (26.96)	1.17 (1.09 ~ 1.26)	<.001	
Q4	1298 (26.13)	1.10 (1.02 ~ 1.18)	0.010	
DBP				0.011
Q1	1024 (20.62)	1.09 (1.00 ~ 1.19)	0.056	
Q2	1432 (28.83)	1.31 (1.21 ~ 1.43)	<.001	
Q3	1225 (24.66)	1.19 (1.09 ~ 1.29)	<.001	
Q4	1286 (25.89)	1.13 (1.05 ~ 1.21)	0.001	

0 1 2
Worse better

b.

Variables	n (%)	OR (95%CI)	P	P for interaction
All patients	4967 (100.00)	1.19 (1.14 ~ 1.24)	<.001	
Age				0.386
Q1	650 (13.09)	1.11 (0.93 ~ 1.31)	0.244	
Q2	1550 (31.21)	1.17 (1.07 ~ 1.27)	<.001	
Q3	1143 (23.01)	1.07 (0.99 ~ 1.16)	0.069	
Q4	1624 (32.70)	1.06 (0.99 ~ 1.13)	0.090	
Waist-hip ratio				0.987
Q1	1239 (24.94)	1.15 (1.06 ~ 1.24)	<.001	
Q2	1244 (25.05)	1.17 (1.07 ~ 1.26)	<.001	
Q3	1242 (25.01)	1.18 (1.09 ~ 1.29)	<.001	
Q4	1242 (25.01)	1.18 (1.08 ~ 1.28)	<.001	
Vital capacity				0.003
Q1	1240 (24.96)	1.22 (1.10 ~ 1.34)	<.001	
Q2	1243 (25.03)	1.26 (1.16 ~ 1.38)	<.001	
Q3	1241 (24.98)	1.14 (1.05 ~ 1.23)	0.002	
Q4	1243 (25.03)	1.04 (0.97 ~ 1.12)	0.303	
Right naked eye vision				0.020
Q1	1109 (22.33)	1.12 (1.03 ~ 1.22)	0.007	
Q2	1228 (24.72)	1.13 (1.04 ~ 1.23)	0.004	
Q3	695 (13.99)	1.08 (0.97 ~ 1.20)	0.168	
Q4	1935 (38.96)	1.28 (1.19 ~ 1.37)	<.001	
Left naked eye vision				0.760
Q1	1028 (20.70)	1.15 (1.05 ~ 1.25)	0.002	
Q2	1150 (23.15)	1.20 (1.10 ~ 1.31)	<.001	
Q3	778 (15.66)	1.13 (1.01 ~ 1.25)	0.026	
Q4	2011 (40.49)	1.18 (1.11 ~ 1.26)	<.001	
BMI				0.037
Q1	1242 (25.01)	1.25 (1.14 ~ 1.37)	<.001	
Q2	1241 (24.98)	1.14 (1.05 ~ 1.23)	0.002	
Q3	1242 (25.01)	1.23 (1.14 ~ 1.34)	<.001	
Q4	1242 (25.01)	1.09 (1.01 ~ 1.18)	0.025	
SBP				0.144
Q1	1216 (24.48)	1.21 (1.10 ~ 1.33)	<.001	
Q2	1114 (22.43)	1.25 (1.14 ~ 1.37)	<.001	
Q3	1339 (26.96)	1.17 (1.08 ~ 1.26)	<.001	
Q4	1298 (26.13)	1.10 (1.03 ~ 1.19)	0.007	
DBP				0.011
Q1	1024 (20.62)	1.11 (1.01 ~ 1.21)	0.030	
Q2	1432 (28.83)	1.33 (1.22 ~ 1.44)	<.001	
Q3	1225 (24.66)	1.18 (1.09 ~ 1.28)	<.001	
Q4	1286 (25.89)	1.13 (1.05 ~ 1.21)	0.001	

0 1 2
Worse better

Figure S2. Stratified associations between sleep debt and lower mental well-being. (**a**) Unadjusted model; (**b**) model adjusted for sex and urban–rural residence.

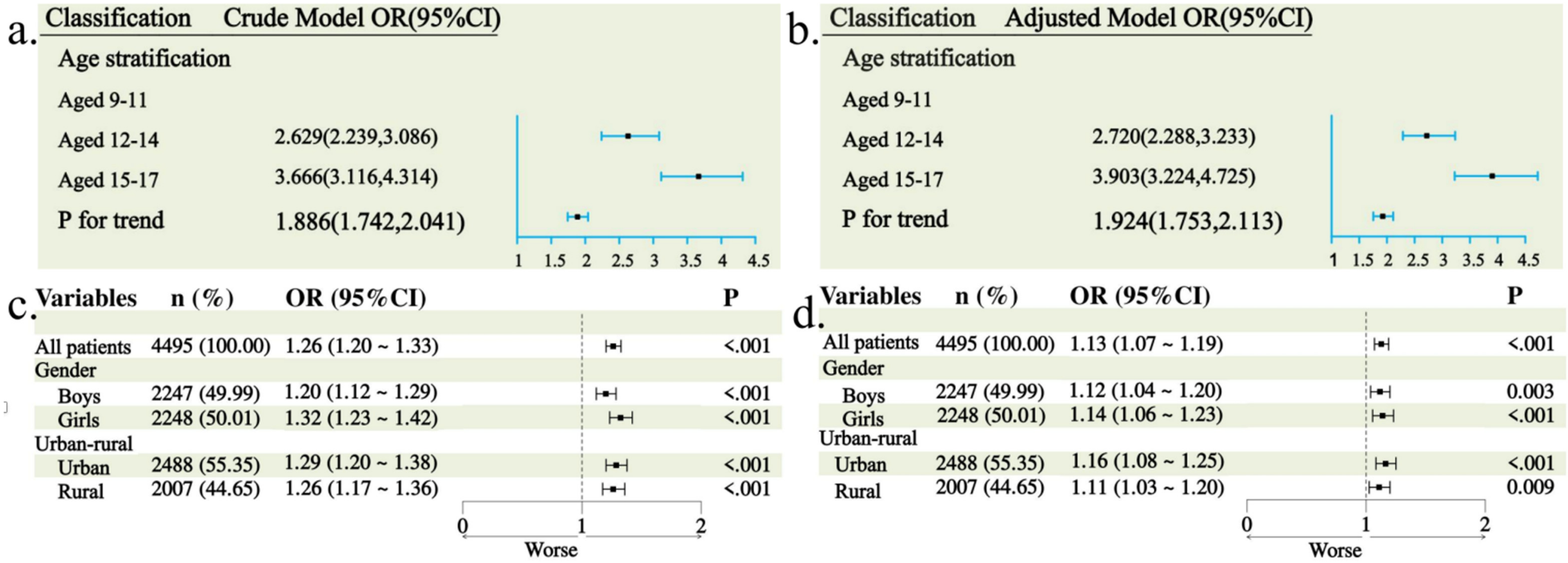

Figure S3. Stratified associations between sleep debt and lower mental well-being after excluding participants with abnormal sleep parameters. (**a**) Age-stratified unadjusted model; (**b**) age-stratified model adjusted for sex, WHR, BMI, and vital capacity; (**c**) unadjusted subgroup model by sex and urban–rural residence; (**d**) model adjusted for age, WHR, BMI, and vital capacity.
